# Lognormal Lorenz and normal receiver operating characteristic curves as mirror images

**DOI:** 10.1098/rsos.140280

**Published:** 2015-02-25

**Authors:** R. John Irwin, Michael J. Hautus

**Affiliations:** School of Psychology, The University of Auckland, Private Bag 92019, Auckland 1142, New Zealand

**Keywords:** Lorenz curve, ROC curve, normal, lognormal

## Abstract

The Lorenz curve for assessing economic inequality depicts the relation between two cumulative distribution functions (CDFs), one for the distribution of incomes or wealth and the other for their first-moment distribution. By contrast, the receiver operating characteristic (ROC) curve for evaluating diagnostic systems depicts the relation between the complements of two CDFs, one for the distribution noise and the other for the distribution of signal plus noise. We demonstrate that the lognormal model of the Lorenz curve, which is often adopted to model the distribution of income and wealth, is a mirror image of the equal-variance normal model of the ROC curve, which is a fundamental model for evaluating diagnostic systems. The relationship between these two models extends the potential application of each. For example, the lognormal Lorenz curve can be used to evaluate diagnostic systems derived from equal-variance normal distributions.

## 2. Introduction

The Lorenz curve of economics and the receiver operating characteristic (ROC) curve of psychology and medicine are closely related analytic tools. The Lorenz curve is primarily used to assess economic inequality, and the ROC curve to evaluate diagnostic systems. In this paper, we review the relation between the two most common forms of these curves—the lognormal model of the Lorenz curve and the normal model of the ROC curve.

The general relation between curves of this kind has been developed by Bamber [1], who did not consider the specific case of Lorenz curves; rather, he reviewed ordinal dominance curves [2], of which Lorenz curves are an important special case. Given two continuous random variables, *X* and *Y* , a point on a graph of an ordinal dominance curve for *X* and *Y* is located at *P*(*X*≤*c*) on the horizontal axis and *P*(*Y* ≤*c*) on the vertical axis. By comparison, a point on a graph of an ROC curve is located at *P*(*X*≥*c*) on the horizontal axis and at *P*(*Y* ≥*c*) on the vertical axis. In both cases, *c* is a constant that can take on values within the domain of *X* and *Y* . Thus, ordinal dominance curves are identical to ROC curves rotated through 180° about the point (1/2,1/2) [1].

Next, consider Lorenz curves as specific examples of ordinal dominance curves. For convenience, we consider continuous distributions only.

## 3. The Lorenz curve

A Lorenz curve that represents the distribution of income or wealth in a society may be defined as a curve ‘…whereby the percentages of the population arranged from the poorest to the richest are represented on the horizontal axis and the percentages of income enjoyed by the bottom *x*% of the population is shown on the vertical axis’ [3, pp. 29–30]. Although Lorenz curves are mostly used to measure inequality in the distribution of income, they have proved valuable analytic tools in other fields as well, including measuring diversity in plant populations and assessing inequality in health among individuals and groups [4,5].

Kendall & Stuart [6] introduce the Lorenz curve by means of a pair of parametric equations in *x*, a positive variable associated with income or other economic quantity. The equations are specified in terms of the probability density function *f*(*t*):

$$F(x)=\int_{0}^{x}f(t)dt$$

and

$$F_{1}(x)=\frac{1}{\mu^{\prime }}\int_{0}^{x}t\;f(t)dt,\;(\mu^{\prime }\neq 0),$$

where *μ*′ is the mean of *f*(*t*).

*F*(*x*) is the cumulative distribution function (CDF) of *X*. The values of the function *F*(*x*) are represented on the horizontal axis of the Lorenz graph. Because people are arranged in order of income, *F*(*x*) shows the cumulative proportion of people with an income less than or equal to *x*. The values of the second function, *F*_1_(*x*) are represented on the vertical axis of the Lorenz graph. Kendall & Stuart [6, p. 48] called this function the ‘incomplete first moment’ distribution. The Lorenz graph plots *F*_1_(*x*) against *F*(*x*), and so depicts the cumulative income accruing to each cumulative proportion of the population. (Although this is the standard procedure for constructing Lorenz curves, in Lorenz's [7] own illustrations of his method he exchanged the vertical and horizontal axes of this description, so that his graphs are in effect rotated versions of those now customarily presented.)

## 4. The receiver operating characteristic curve

The ROC curve evaluates how well a diagnostic system can distinguish between two events, such as how well a glucose tolerance test differentiates the presence from the absence of diabetes, or how well a psychological checklist differentiates potential recidivists from law-abiding persons. ROC curves have many other applications, including statistical analysis (e.g. [8]). In view of the provenance of ROC analysis in detection theory [9], the events are often designated ‘signal’ and ‘noise’ and abbreviated ‘s’ and ‘n’. The vertical axis of an ROC graph is usually called the hit rate, and, as is well known, it is a conditional probability for correctly distinguishing between two events, A and B. It is the probability that, given that Event A occurred, Event A was correctly identified as having occurred. We denote this conditional probability as *P*_H_. Similarly the horizontal axis of an ROC graph is often called the false-alarm rate. It is the conditional probability that, given that Event B occurred, it was mis-identified as Event A. We denote this probability as *P*_F_. (The other two possible outcomes—misses and correct rejections—are complements of hits and false-alarms, and so provide no additional information.)

Parametric equations for the ROC are

$$P_{H}(c)=1-\int_{-\infty }^{c}f_{s}(x)dx$$

and

$$P_{F}(c)=\int_{-\infty }^{c}f_{n}(x)dx,$$

where *f*_*s*_(*x*) and *f*_*n*_(*x*) are probability density functions associated with s and n, respectively, and the criterion *c* is set by the diagnostic system. Note that the integration limits depend on the domain of the distribution assumed.

## 5. An equation for the lognormal Lorenz curve

The equation for the Lorenz curve, *L*(*p*), is

$$L(p)=F_{1}(x),$$

where

p=F(x)

and where 0≤*p*≤1.

To find a function for the lognormal Lorenz curve, a suitable parametrization of the lognormal distribution is required. The two-parameter version has two properties that commend it for modelling income: it is valid only for positive values, and it has a long upper tail, both of which usually characterize the distribution of income.

The lognormal distribution is so named because it is a distribution whose logarithm is normally distributed. If Y=ln⁡(X) is a normally distributed random variable with two parameters (mean=*μ* and variance=*σ*^2^), then *X* is said to be lognormally distributed with two parameters, *μ* and *σ*^2^. The probability density function for a two-parameter lognormal distribution [10, eqn (2.5)] is

$$f(x)=\frac{1}{x\;\sigma \sqrt{2\pi }}\exp\left(-\frac{{\left(\ln(x)-\mu \right)}^{2}}{2\sigma^{2}}\right).$$

The lognormal CDF with parameters in logarithmic space of *μ* and *σ*^2^ can be written as

5.1 $$F(x)=\Phi \left(\frac{\ln(x)-\mu }{\sigma }\right)\;\mathrm{with}\;x>0,$$

where *Φ*(⋅) is the normal distribution function. The CDF for the first-moment distribution of the lognormal distribution is given by the theorem [10] that the *j*th moment distribution function of a lognormal distribution with parameters *μ* and *σ*^2^ is itself a lognormal distribution with parameters *μ*+*jσ*^2^ and *σ*^2^, where here *j*=1. Thus

5.2 $$F_{1}(x)=\Phi \left(\frac{\ln(x)-(\mu +\sigma^{2})}{\sigma }\right).$$

An equation for the lognormal Lorenz curve can be obtained by solving equation (5.1) for ln⁡(x) followed by substitution into equation (5.2), thereby eliminating *x*. Rearrangement of equation (5.1) yields

$$\ln(x)=\sigma \;\Phi^{-1}(p)+\mu ,$$

which, when substituted for ln⁡(x) in equation (5.2), gives

$$L(p)=\Phi \left(\frac{\left[\sigma \Phi^{-1}(p)+\mu \right]-(\mu +\sigma^{2})}{\sigma }\right),$$

and simplifies to

5.3 $$L(p)=\Phi (\Phi^{-1}(p)-\sigma )$$

This result can be found in, for example, Cowell [11, p. 157].

## 6. An equation for the equal-variance normal receiver operating characteristic curve

Perhaps, the most common model of the ROC curve is that based on two equal-variance normal distributions, one distribution for the noise and the other for the signal plus noise. The origin of the distributions is usually assigned to the mean of the noise distribution. The scale factor is set by assigning unit value to each variance. As a result, the ROC curve has only one parameter, namely *d*′, the mean of the signal distribution specified in standard deviation units.

Whereas the axes of the Lorenz curve are CDFs, those of the ROC curve are complements of CDFs. Thus, if *P*_F_ is computed from a normal distribution with mean 0 and variance 1, then

6.1 $$P_{F}=1-\Phi (c).$$

Similarly, if *P*_H_ is computed from a normal distribution with mean *d*′ and variance 1, then

6.2 $$P_{H}=1-\Phi (c-d^{\prime }).$$

We write the equation for the ROC curve, *R*(*p*), as

$$R(p)=P_{H}(c),$$

where *p*=*P*_F_ and again 0≤*p*≤1. Then inverting equation (6.1) gives

$$c=\Phi^{-1}(1-P_{F})$$

and substituting the result into equation (6.2) to eliminate *c* yields

$$R(p)=1-\Phi (\Phi^{-1}(1-P_{F})-d^{\prime }),$$

which simplifies to

6.3 $$R(p)=\Phi (\Phi^{-1}(P_{F})+d^{\prime }).$$

## 7. Relation between the lognormal Lorenz and the equal-variance normal receiver operating characteristic curves

Observe that equation (6.3) for the equal-variance normal ROC curve is the same as equation (5.3) for the lognormal Lorenz curve but with *d*′ in place of −*σ*. Furthermore, if *μ* is the mean of a lognormal density in logarithmic space, then *μ*+*σ*^2^ is the mean of its first-moment density [10]. Recall that *d*′ is the difference between the means of these two normal densities divided by their common standard deviation, so

$$d^{\prime }=\frac{[\mu +\sigma^{2}]-\mu }{\sigma }=\sigma .$$

Hence the parameters *d*′ and *σ* in equations (5.3) and (6.3) are equal but opposite in sign.

Equations (5.3) and (6.3) are important examples of the proof that the ordinal dominance curve and its corresponding ROC curve are related by a 180° rotation about the point (1/2,1/2) [1]. To confirm this for equations (5.3) and (6.3), note that the equations for rotating a point through 180° around the point (1/2,1/2) are *x*′=1−*x* and *y*′=1−*y* (e.g. Dodge [12]). Substituting these values into equation (5.3) for the lognormal Lorenz yields

$$1-y=\Phi (\Phi^{-1}(1-p)-\sigma ),$$

which simplifies to

$$y=\Phi (\Phi^{-1}(p)+\sigma )$$

and is the same as equation (6.3) for the equal-variance ROC curve.

The lognormal Lorenz curve and the equal-variance normal ROC curve are thus congruent. In addition, Aitchison & Brown [10] observed that the lognormal Lorenz curve is symmetrical about the negative diagonal of a unit square, that is about the line drawn from the point (0, 1) to (1, 0). Similarly, the equal-variance normal ROC curve is symmetrical about the negative diagonal [13, p. 60]. The congruence of the two curves and their symmetry about the negative diagonal means that, after a rotation through 180° about the point (1/2,1/2), the rotated and original curves appear to be mirror images in the positive diagonal of the square.

Kakawani [14] listed some conditions that Lorenz curves satisfy: *L*(0)=0; *L*(1)=1; *L*′(*p*)≥0; *L*′′(*p*)≥0 and *L*(*p*)≤*p*. The equal-variance normal ROC satisfies similar conditions: *R*(0)=0; *R*(1)=1; *R*′(*p*)≥0; *R*′′(*p*)≤0 and *R*(*p*)≥*p*.

## 8. Indices of the lognormal Lorenz and the equal-variance normal receiver operating characteristic curves

**Figure 1.**
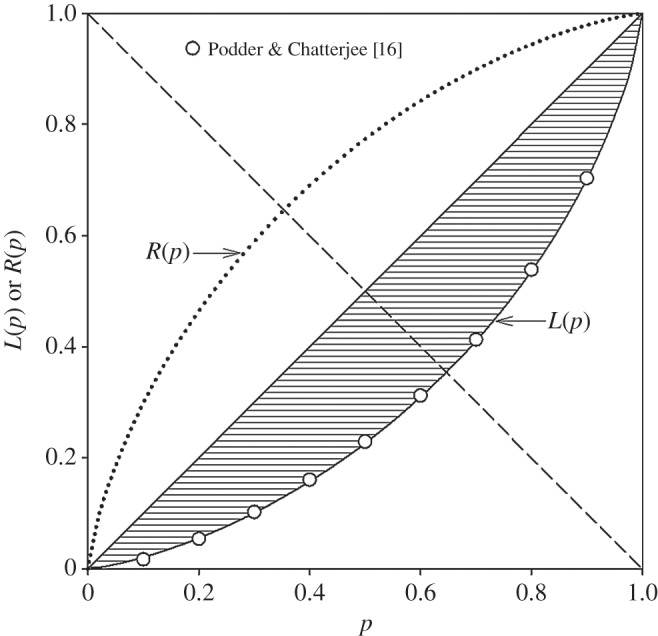
A lognormal Lorenz curve *L*(*p*) for *σ*=0.751 fitted to Podder & Chatterjee's data [16] for the distribution of New Zealand household income in 1995/1996. The hatching shows the area of concentration. The equal-variance normal ROC curve *R*(*p*) for *d*′=0.751 is shown for comparison. Each curve is symmetrical with respect to the negative diagonal (dashed line).

An important summary index for ROC curves is the area under the curve. The area under an equal-variance normal ROC curve can be computed from its detection-theoretic index of accuracy *d*′. It is given by the equation $A_{R}=\Phi (d^{\prime }/\sqrt{2})$, where *A*_*R*_ is the proportion of the area under the curve [15]. For example, the area under the ROC curve with *d*′=0.752, illustrated in figure 1, is 0.702. The area between the ROC curve and the positive diagonal is *A*_*R*_−0.5, which for the example in figure 1 is 0.202. This is also the proportion of the square's area between the diagonal and the lognormal Lorenz curve (the hatched area in figure 1), which is an important quantity in economics known as the area of concentration. Kendall & Stuart [6, p. 49] proved that the standard index of inequality, the Gini coefficient of concentration (0≤*G*≤1), is twice the area of concentration.

The indices *d*′ and *G* for the equal-variance normal ROC curves and for lognormal Lorenz curves are related. From geometry it is clear that *G*=2(*A*_*R*_−0.5)=2*A*_*R*_−1. Furthermore, because $A_{R}=\Phi (d^{\prime }/\sqrt{2})$, it follows that $G=2(\Phi (d^{\prime }/\sqrt{2})-0.5)=2\Phi (d^{\prime }/\sqrt{2})-1$. Recall that for the lognormal Lorenz, *d*′=(*μ*+*σ*^2^−*μ*)/*σ*=*σ*. Substituting *σ* for *d*′ gives $G=2\Phi (\sigma /\sqrt{2})-1$, which is a standard equation for computing *G* for the lognormal Lorenz (e.g. [10]).

## 9. An empirical example

Podder & Chatterjee [16] examined changes in the distribution of New Zealand household income from 1984 to 1996. They provided the ordinate values corresponding to each decile of income, so that graphs of Lorenz curves can be constructed from their numerical results. As an example, figure 1 shows their data for 1995/1996 (their table 2). The solid smooth curve in figure 1 is our estimate of the best-fitting lognormal Lorenz curve to those data. The fit was determined using SDT Assistant software [17] to fit the equal-variance normal ROC to confidence ratings. The ROC parameter *d*′, or equivalently the Lorenz parameter *σ*, for the fitted curve is 0.751. Podder & Chatterjee [16] reported a Gini coefficient of 0.404 for these data. The Gini coefficient for the fitted curve in figure 1 is 0.405 which attests to the closeness of the fit of the lognormal model to the data. The mirror image ROC curve for *d*′=0.751 is shown in figure 1 for comparison.

## 10. Discussion

The preceding comparison of lognormal Lorenz and equal-variance normal ROC curves has described how they are congruent and related by a 180° rotation about the point (1/2,1/2). That comparison neglects an important feature of ROC analysis that concerns the criterion or threshold for a decision. In many applications of ROC analysis, both the location of the criterion and the accuracy of the system under test are of interest. As is well known, the location of a criterion is shown by a point on the ROC curve, whereas the accuracy of the system is specified by some index that characterizes the curve as a whole.

For the Lorenz curve, by contrast, the location of the criterion is normally not of primary interest because its main purpose is to provide a summary index of inequality. Like ROC analysis, it yields that index on the basis of the curve as a whole, from which the area of concentration and the Gini coefficient of inequality can be determined. To obtain that result, analysts do not need to decide, for example, what level of income constitutes poverty. The Gini index of the Lorenz curve thus measures a dimension analogous to accuracy of the ROC curve and yields a measure that is independent of decision criteria. Of course, analysts may be interested in particular points on a Lorenz curve, such as the point showing what percentage of the population earns 80% of the total income, but these points are not interpreted, as they are in ROC analysis, as indicating the diagnostic system's choice of decision criteria.

Nonetheless, an analysis confined to comparing the shapes of Lorenz curves and the shapes of their 180° rotated ROC curves does not take into account the effect of such a rotation on the location of corresponding points on each curve. The Lorenz point (*x*,*y*) and the 180° rotated ROC point (1−*x*, 1−*y*) are not mirror images, though they may lie on curves that are mirror images, such as the lognormal Lorenz curve and the equal-variance normal ROC curve.

One benefit of this relation is that readily available ROC software is equally applicable to the analysis of Lorenz curves. In addition, the potential applications of each curve are extended. For example, the lognormal Lorenz curve could be used to evaluate diagnostic systems that might otherwise be modelled by the equal-variance normal ROC. And the measurement of economic inequality can be added to the large repertoire of existing applications of equal-variance normal ROC analysis.

Our analysis deals only with equal-variance ROCs. Lee [18] compared Lorenz and unequal-variance ROC curves in the evaluation of diagnostic tests. He observed that unequal ROC curves can be constructed for diagnostic tests that are not monotonic with likelihood ratio, in which case the ROC curves may dip below the positive diagonal of the unit square. Such ROC curves are sometimes called ‘improper’. Lorenz curves cannot be improper in this sense because they are by definition convex. In other words, they satisfy the condition, noted above, that *L*′′(*p*)≥0. Thus, improper ROC curves do not have a corresponding rotated Lorenz curve. To construct such a Lorenz curve, the results of the diagnostic test have to be re-ordered to be monotonic with likelihood ratio. Lee's examples illustrate that the general relation between ROC and Lorenz curves does not extend to improper ROC curves.

## 11. Conclusion

Although Lorenz and ROC curves stem from entirely different origins and have been applied to entirely different problems, they are intimately connected. In particular, the equations for two important examples—the lognormal Lorenz curve and the equal-variance ROC curve—are identical except for the sign of their respective parameters. Moreover, these symmetric Lorenz and ROC curves are congruent and are mirror images in the positive diagonal. Because of this close relationship, the potential areas of application of each curve are broadened.

## Data accessibility

The data in figure 1 are available from Podder & Chatterjee [16, table 2].
